# Study of Inflammatory Bowel Disease in Patients Undergoing Colonoscopy at a Tertiary Center of Nepal

**DOI:** 10.7759/cureus.67045

**Published:** 2024-08-16

**Authors:** Kumud Bhattarai, Ajit Khanal, Ramila Shrestha, Mukesh S Paudel

**Affiliations:** 1 Gastroenterology, National Academy of Medical Sciences, Kathmandu, NPL

**Keywords:** crohn's disease, ulcerative colitis, colonoscopy, incidence rate, inflammatory bowel disease

## Abstract

Introduction

Inflammatory bowel disease (IBD) is a chronic inflammatory condition of the gastrointestinal tract that includes ulcerative colitis (UC) and Crohn’s disease (CD). The incidence and prevalence of disease are on a rising trend. Increased case detection is related to better health awareness and improved availability of diagnostic services in the community. This article aims to calculate the incidence of IBD per 1,000 colonoscopies per year and to study the clinical and demographic characteristics of patients with IBD.

Methodology

It was a prospective observational study done at the National Academy of Medical Sciences, Bir Hospital, Kathmandu, Nepal, from March 2023 to February 2024. All the patients who underwent colonoscopy during the study period were assessed for possible diagnosis of IBD. The Incidence rate was calculated as new IBD cases per 1,000 colonoscopies per year. Demographic and clinical profiles of the patients were collected.

Results

Among 1,248 patients who underwent colonoscopy during the study period, IBD was detected in 52 patients (4.16%). UC was diagnosed in 43 patients and the incidence rate of it was calculated to be 34.4 cases per 1,000 colonoscopies per year. Similarly, CD was diagnosed in nine patients with its incidence rate being 7.2 cases per 1,000 colonoscopies per year. Disease was predominantly seen in females (F:M ratio - 1.36:1). The mean age at diagnosis of IBD was 39.67 ± 14.53 years, with patients with CD being slightly younger than those with UC. The majority of patients with UC had pancolitis. The median time to diagnosis from symptom onset was three years (range: 6 months to 7 years) for CD and 10 months for patients with UC (range: 2 months to 5 years). The most common extra-intestinal manifestations were arthralgia (11, 21.15%) and arthritis (7, 13.46%). Traditional drugs like mesalamine, prednisolone, and azathioprine were commonly prescribed. Biologics were used only in two (3.84%) patients, including infliximab and adalimumab. Janus kinase inhibitor (tofacitinib) was used in three (5.76%) patients in cases of acute severe UC.

Conclusions

Due to the rising trend of awareness in health and availability of colonoscopy services in our country like Nepal, the incidence of IBD is seen to be quite high. UC was seen more commonly than CD, and females were predominantly involved. The majority of patients belonged to the young and middle-aged population. The majority of patients with UC had extensive colitis, while patients with CD had ileo-colonic disease with non-stricturing, non-penetrating phenotypes. Arthralgia and arthritis were the most common extra-intestinal manifestations. Conventional drugs like mesalamine, prednisolone, and immunomodulators such as azathioprine were mostly used. The use of biologics was fairly low. This study certainly contributes to the existing literature from Nepal regarding IBD.

## Introduction

Inflammatory bowel disease (IBD) is a chronic immune-mediated inflammatory condition of the gastrointestinal tract that consists of two entities: Crohn’s disease (CD) and ulcerative colitis (UC). It has a relapsing and remitting course. UC is mostly limited to the colon with inflammation starting at the rectum as proctitis and extending proximally in a continuous manner. CD mainly involves distal ileum and colon but any part of the gastrointestinal tract from the oral cavity to the anus may be involved [1]. A complex interplay between environmental factors in genetically susceptible hosts and gut microbiota is mainly implicated in the pathogenesis of IBD [2]. Previously, IBD was believed to be a disease of the Western world. However, the increasing incidence of IBD in Asian and African countries suggests that westernization of lifestyle and urbanization may play key influences in acquiring IBD [3].

Ontario (Canada) is believed to have the highest prevalence of IBD in the world with a standardized prevalence of 534.3 per 100,000 people [4]. Other Western countries with a high prevalence of IBD include Norway and Germany [5]. Among Asian countries, the highest incidence of UC is seen in India (6/100,000 person-year), and the highest incidence of CD in Korea (5.1/100,000 person-year) [6]. A population-based prospective study from India found an overall prevalence of UC of 44.3/100,000 population [7].

Two cases of IBD from Nepal, one each of CD and UC were documented in English literature by Probert et al. in 1990 in patients who presented with bloody diarrhea attending mission hospitals in the Indian subcontinent [8]. Another case of CD was reported from Nepal only in 2009 in a resected sample of ileum who presented with perforation peritonitis [9]. In a study done by Paudel et al. from Nepal, which was a retrospective observational study, reported the incidence of UC as 23.7 per 1,000 colonoscopies per year and the incidence of CD as 1.6 per 1,000 colonoscopies per year [10].

Very few studies have been published in Nepal regarding the epidemiology and clinical characteristics of patients with IBD. We aim to calculate the incidence of IBD per 1,000 colonoscopies per year and study the clinical and demographic characteristics of patients with IBD. This study certainly contributes to the existing literature from Nepal regarding IBD.

## Materials and methods

This prospective observational study was done at the National Academy of Medical Sciences, a tertiary-level referral center in Nepal. Approval was taken from the Institutional Review Board (Ref no. 774/2079/80). All the patients >16 years of age who underwent colonoscopy from March 2023 to February 2024 were assessed for possible diagnosis of IBD. The diagnosis was based on clinical presentation, endoscopic features, radiological examination when required; and confirmed with histology. UC was suspected in colonoscopy with features of loss of vascular pattern, granularity of mucosa, erythema, erosions, ulcerations, and pseudopolyps. It was confirmed on histology with mucosal inflammatory cells particularly lymphocytes infiltrating into the lamina propria, architectural distortion of crypts, crypt abscess, and the hallmark being the absence of granulomas. Similarly, the CD was suspected in colonoscopy based on discontinuous skip lesions, aphthous ulcerations, cobblestone appearance, and ileal involvement. Similarly, the radiological features include skip lesions, strictures, and fistulizing disease. Histologic confirmation was based on the presence of chronic inflammation, crypt distortion, and its hallmark being noncaseating granuloma [11]. The phenotypic distribution of CD was based on the Montreal classification that includes: age at diagnosis, disease location, disease behavior, and perianal disease modifier if present. Similarly, the extent of colonic involvement in UC was based on the Montreal classification which includes E1 disease (proctitis), E2 disease (left-sided colitis), and E3 disease (extensive colitis) [12].

The cases of IBD that were diagnosed before the study duration were excluded and only newly diagnosed cases were included. All the demographic characteristics, and clinical features including appendectomy status, prior bowel surgery, extra-intestinal manifestations, and the prescription pattern of drugs were recorded in a proforma. Similarly, the smoking status of patients was enquired and recorded.

**Figure 1:**
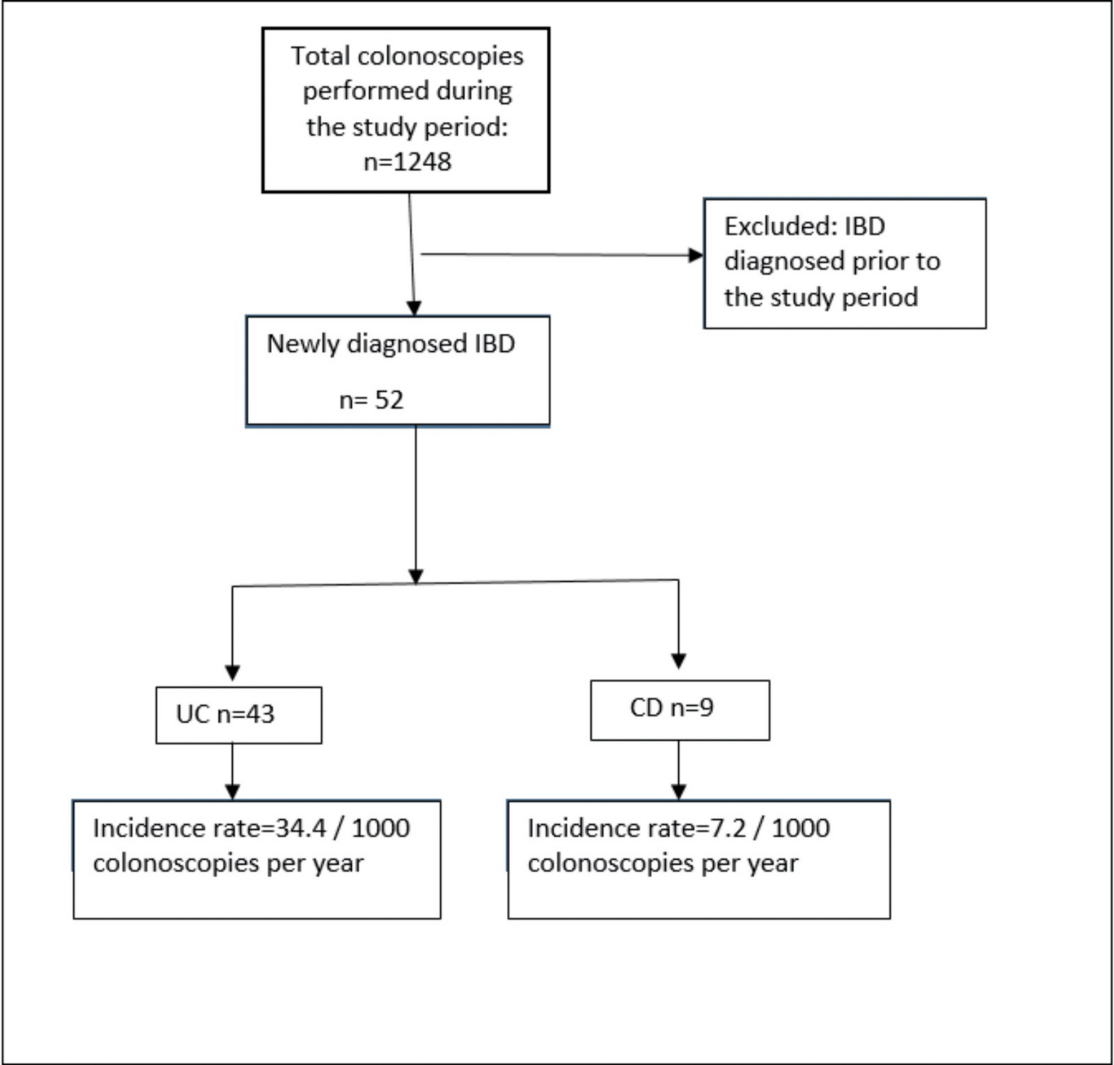
Study flow diagram. Image credit: All authors. IBD, inflammatory bowel disease; UC, ulcerative colitis; CD, Crohn's disease

Data were entered in SPSS Version 16 software (SPSS Inc., Chicago, IL) and analyzed. Continuous variables were expressed as mean ± standard deviation (SD) or median (range) based on their distribution. Similarly, categorical data were expressed as frequency and percentage when appropriate. The denominator for the incidence rate was taken as total colonoscopies performed in a year, and the incidence rate was expressed as new cases per 1,000 colonoscopies per year for UC and CD. The flow diagram of the study is shown in Figure 1.

## Results

A total of 1,248 patients who underwent colonoscopy during the study period were included in the study. IBD was diagnosed in 52 cases (4.16%). UC was seen in 43 patients and its incidence rate was calculated to be 34.4 per 1,000 colonoscopies per year. Similarly, CD was diagnosed in nine patients with its incidence rate being 7.2 cases per 1,000 colonoscopies per year.

**Figure 2:**
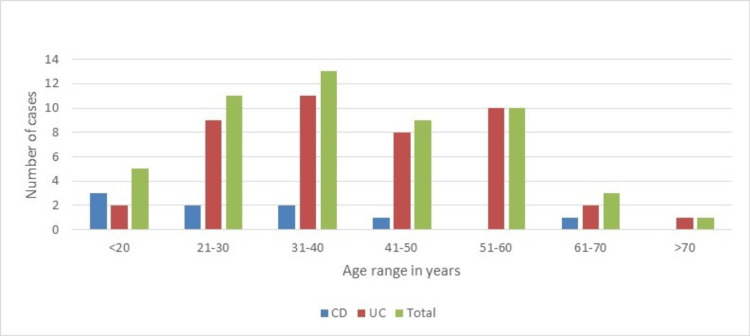
Age distribution of patients with IBD. IBD, inflammatory bowel disease; UC, ulcerative colitis; CD, Crohn's disease

The mean age of patients at diagnosis of IBD was 39.67 ± 14.53 years. Patients with CD were slightly younger (35.80 ± 16.28; range: 17-69 years) than those with UC (42.56 ± 14.3; range: 19-72 years). There was a female predominance with a female:male ratio of 1.36:1. The age distribution of patients is shown in Figure 2.

The majority of patients were without any formal education (13, 25%) or only with a primary level of education (12, 23.07%), both combined, comprising nearly half of the total cases (Table 1).

**Table 1:** Clinical characteristics of patients with IBD. IBD, inflammatory bowel disease; UC, ulcerative colitis; CD, Crohn's disease; SD, standard deviation

	Number of patients with UC, *n* (%)	Number of patients with CD, *n* (%)	Total, *n* (%)
Age (years ± SD)	42.56 ± 14.3	35.80 ± 16.28	39.67 ± 14.53
Sex (Female:Male)	1.38:1	1.25:1	1.36:1
Education			
Primary level	11 (25.58%)	1 (11.11%)	12 (23.07%)
High school	15 (34.88%)	6 (66.66%)	21 (40.38%)
Graduation and above	5 (11.62%)	1 (11.11%)	6 (11.53%)
No formal education	12 (27.90%)	1 (11.11%)	13 (25.0%)
Residence			
Rural	27 (62.79%)	3 (33.33%)	30 (57.69%)
Urban	16 (37.21%)	6 (66.66%)	22 (42.30%)
Smoking status			
Current	1 (2.32%)	0	1 (1.92%)
Former	9 (21%)	1 (11.11%)	10 (19.23%)
Never	33 (76.74%)	8 (88.88%)	41 (78.84%)
Appendectomy	0	0	0
Bowel resection	0	2 (22.22%)	2 (3.84%)
Median duration of symptoms till diagnosis	10.5 months	3 years	

**Figure 3:**
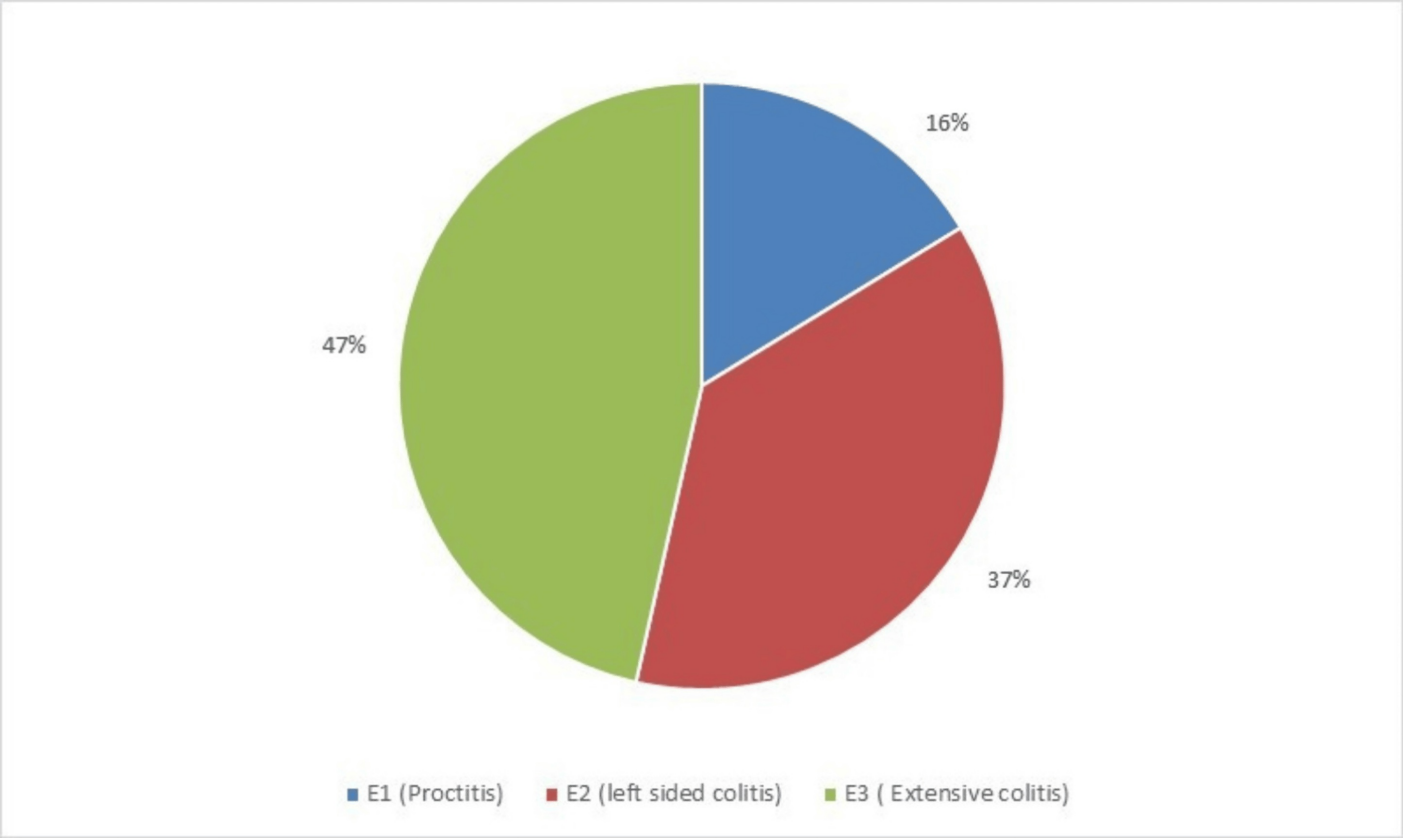
Disease extent of ulcerative colitis.

The median delay in symptom onset and diagnosis was 10 months in patients with UC (range: 2 months to 5 years) and 3 years (range: 6 months to 7 years) in CD. Regarding disease extent in UC, the majority had extensive colitis (20, 46.52%) and left-sided colitis (16, 37.20%) (Figure 3).

The majority of patients with CD had L3 (ileo-colonic) disease with B1 ( non-stricturing, non-penetrating) phenotype (Table 2).

**Table 2:** Montreal classification of patients with IBD. IBD, inflammatory bowel disease; UC, ulcerative colitis; CD, Crohn's disease

Parameters	Number of patients with UC, *n* (%)	Number of patients with CD, *n* (%)
Age at diagnosis (years)		
A1 (<16)	0	0
A2 (17-40)	22 (51.16%)	7 (77.77%)
A3 (>40)	21 (48.83%)	2 (22.22%)
Disease location		
L1 (Terminal ileal )	_	2 (22.22%)
L2 ( colonic)	_	1 (11.11%)
L3 ( Ileocolonic)	_	6 (66.66%)
L4 ( Isolated upper bowel)	_	
E1 (Proctitis)	7 (16.28%)	_
E2 (Left-sided colitis)	16 (37.20%)	_
E3 (Extensive colitis)	20 (46.52%)	_
Disease behavior		
B1 (Non-stricturing, non-penetrating)	_	5 (55.55%)
B2 (Stricturing)	_	2 (22.22%)
B3 (Penetrating)	_	2 (22.22%)

Per-rectal bleeding (43, 100%) was the commonest presenting symptom in patients with UC. Other symptoms included tenesmus (35, 81.39%), chronic diarrhea (24, 55.81%), and mucous discharge (23, 53.48%). Pain abdomen (9, 100%) and weight loss (6, 66.66%) were the predominant presenting symptoms in CD. Other features of this disease included chronic diarrhea and fever (Table 3).

**Table 3:** Presenting symptoms of patients with IBD. IBD, inflammatory bowel disease; UC, ulcerative colitis; CD, Crohn's disease

Symptoms	Number of patients with UC, *n* (%)	Number of patients with CD, *n* (%)
Per-rectal bleed	43 (100%)	0
Mucous discharge	23 (53.48%)	1 (11.11%)
Tenesmus	35 (81.39%)	0
Chronic diarrhea	24 (55.81%)	4 (44.44%)
Weight loss	3 (6.97%)	6 (66.66%)
Pain abdomen	4 (9.30%)	9 (100%)
Fever	1 (2.32%)	2 (22.22%)

**Figure 4:**
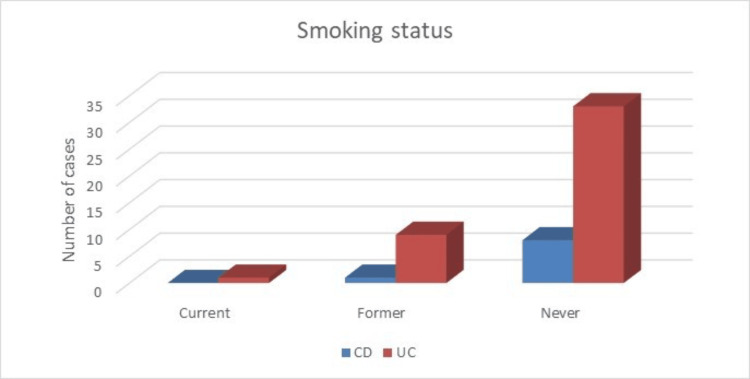
Smoking status of patients with IBD. IBD, inflammatory bowel disease; UC, ulcerative colitis; CD, Crohn's disease

Arthralgia (11, 21.15%) and arthritis (7, 13.46%) were the commonest extra-intestinal manifestations. These features were predominantly seen in patients with CD than in those with UC. Other extra-intestinal manifestations were less frequent in our study (Table 4). One-fifth of patients with UC were former smokers, whereas none of the patients with CD were active smokers (Figure 4).

**Table 4:** Extra-intestinal manifestations of patients with IBD. IBD, inflammatory bowel disease; UC, ulcerative colitis; CD, Crohn's disease

Features	Number of patients with UC, *n* (%)	Number of patients with CD, *n* (%)	Total, *n* (%)
Arthralgia	7 (16.27%)	4 (44.44%)	11 (21.15%)
Arthritis	4 (9.30%)	3 (33.33%)	7 (13.46%)
Erythema nodosum	1 (2.32%)	0	1 (1.92%)
Uveitis	0	1 (11.11%)	1 (1.92%)
Episcleritis	0	0	0
Primary sclerosing cholangitis	0	0	0

Oral mesalamine (42, 97.67%) and prednisolone (27, 62.79%) were the commonest drugs prescribed in UC. The majority of the patients with UC received 5-ASA suppositories (20, 46.51%) as local therapy. Similarly, azathioprine was used at least as the starting treatment in 100% of the patients with CD. Biologics were used only in two (3.84%) of the total IBD patients, which included infliximab and adalimumab. Janus kinase inhibitor in the form of tofacitinib was used in three patients (5.76%) with acute severe UC (Table 5).

**Table 5:** Prescription pattern in patients with IBD. IBD, inflammatory bowel disease; UC, ulcerative colitis; CD, Crohn's disease; 5-ASA, 5-aminosalicylic acid

Drugs	Number of patients with UC, *n* (%)	Number of patients with CD, *n* (%)	Total, *n* (%)
Mesalamine	42 (97.67%)	4 (44.44%)	46 (88.46%)
Sulfasalazine	1 (2.32%)	0	1 (1.92%)
Prednisolone	27 (62.79%)	5 (55.55%)	32 (61.53%)
Budesonide	1 (2.32%)	1 (11.11%)	2 (3.84%)
Azathioprine	5 (11.62%)	9 (100%)	14 (26.92%)
Tofacitinib	3 (6.97%)	0	3 (5.76%)
Infliximab	0	1 (11.11%)	1 (1.92%)
Adalimumab	0	1 (11.11%)	1 (1.92%)
Local therapy			
5-ASA Suppository	20 (46.51%)	0	
5-ASA Enema	1 (2.32%)	0	

## Discussion

IBD is a chronic immune-mediated inflammatory condition with global prevalence. It has four different epidemiological stages including the stage of emergence, acceleration in incidence, compounding prevalence, and prevalence equilibrium. Developing countries fall in the stage of emergence whereas many countries of the Western world are in the stage of compounding prevalence [13]. The incidence and prevalence of IBD were low in Asia a few decades back. However, the case detection rate has increased due to various factors, including industrialization, access to healthcare facilities, and improved diagnostic modalities [3]. Most of our patients were in their thirties and forties, who are the active group of the population. The chronic nature of the disease poses a substantial public health challenge in the community.

Studies on the epidemiology of IBD have shown a bimodal age distribution with disease peaking at 20-40 years and next peak slightly later, at 60-80 years. The second peak is more often seen in UC than Crohn’s. However, in the Asian population, the second peak is less pronounced [14]. In a few studies done from Nepal in a similar setting, the disease was predominantly seen in the age group of 20-45 years and the second peak was not observed as in the Western population [10,15]. Our study also showed a predominance of disease in 20-40 years, with the absence of a second peak. The true prevalence of IBD has not been well studied in Nepal. The reasons behind this are the lack of population-based studies, and the country lacking its own IBD registry. Our study showed the incidence of UC to be 34.4 per 1,000 colonoscopies per year and that of CD to be 7.2 cases per 1,000 colonoscopies per year. The incidence rate calculated is higher than the previous study done a few years back by Paudel et al. in Nepal [10].

Abhirami et al., in their study in India, revealed that IBD was more prevalent in the male population similar to the West [16]. Our study revealed a female predominance with a female: male ratio of 1.35:1. In a retrospective study by Paudel et al. in Nepal, there was a male predominance of disease with a male: female ratio of 1.4:1 in UC and 1.7:1 in CD [10]. Another study by Pathak et al. in Nepal showed an almost similar incidence of UC between both sexes with a male-to-female ratio of 1.04:1 [15]. The female predominance in our study may be due to it being a single-center study and relatively fewer cases in comparison to a previously done study by Paudel et al. [10]. The majority of patients in our study resided in rural areas (57.69%). Although IBD is considered a disease of urbanization, our finding of higher prevalence of IBD amongst people of rural areas may be because of the adoption of a Western lifestyle including food habits even in rural areas or other related genetic factors.

A delay in diagnosis in patients with IBD may be attributed to the disease mostly being a low-grade chronic inflammation, some patients presenting only with subtle symptoms or patients often underestimating their symptoms. Yewale et al. showed a median delay of 12 months duration from symptom onset to diagnosis for UC and 24 months for CD [17]. In our study, the median delay in symptom onset and diagnosis was 10 months (range: 2 months to 5 years) in UC and three years (range: 6 months to 7 years) in CD. Factors that might be associated include ignorance of the disease and poor availability of healthcare services in that particular community. Intestinal tuberculosis being the great mimicker of CD, the patients of CD might be initially treated with antitubercular therapy, which might lead to some delay in diagnosis in the case of CD. Four out of nine patients (44.44%) were initially treated with anti-tubercular therapy before a definitive diagnosis of CD was made. In our study, the majority of patients with IBD had either no formal education (25%) or only primary-level education (23.07%), both combined, comprising nearly half of the total cases. This is in contrast to the study done by Yewale et al. in India, where the majority of the patients (55%) of IBD were either graduates or postgraduates [17]. This difference may be related to the preexisting low level of education of the study community and the state. Educational background is particularly important in IBD regarding compliance and affordability of the treatment [18]. The common extra-intestinal manifestations (EIM) in our study were arthralgia and arthritis. The EIM ranged from 10.5% to 35% in different studies done in Nepal [19,20]. In one survey done in India, one or more of the EIM was seen in more than fifty percent of the patients, the commonest being arthralgia with or without arthritis [ 21]. The occurrence of EIM might still be less in our study because only the newly diagnosed cases were considered.

Treatment of UC and CD consists of phases of induction of remission and maintenance of remission. The emergence of biologics and small molecules have revolutionized the treatment of IBD, and are considered a cornerstone in the management of moderate-severe forms of disease [ 22]. Treat-to-target strategies have been proposed by selecting therapeutic targets in the IBD ( STRIDE II) initiative. These targets include clinical remission, endoscopic remission, and normalization of inflammatory biomarkers including C-reactive protein, erythrocyte sedimentation rate, and fecal calprotectin. Histologic healing and transmural healing are not kept as the formal targets [23]. The majority of patients in our study were prescribed drugs including mesalamine, prednisolone, and immunomodulatory agents in the form of azathioprine. Hundred percent of patients with CD received azathioprine whereas only 11% of those with UC received it. The use of biologics was fairly low (3.84%) which included infliximab and adalimumab which were used in patients with CD. In the study done in India by Makharia et al., 99.6% of patients with UC, and 95.6% of patients with CD never used biologics therapy [21]. In the era of biologics, the cost and easy availability of drugs are the major concern. The only available biologics/biosimilars and small molecules in Nepal are adalimumab (a TNF-α inhibitor), and tofacitinib (a Janus kinase inhibitor). The cost of biologics is high and is not affordable to many patients. The treatment cost of TNF-α antibody in neighboring country India ranged from 20,000 to 22,000 US$ annually a few years ago [24]. The cost of biologics seems to be gradually decreasing in recent years. In a study of cost variance analysis of biological drugs marketed in India, the cost of 40 mg of adalimumab injection ranged from 194.29 to 302.22 US$, and 100 mg of infliximab injection ranged from 386.85 to 496.12 US$ [25]. Reducing the cost of biologics/biosimilars and making them readily available in lower-middle-income countries like Nepal is of utmost concern.

The major limitation of this study is that it was conducted in a single center for only one year. For a more comprehensive evaluation of the epidemiological and other clinical aspects of the disease, a multicenter study with a longer duration is necessary. The denominator of the incidence rate which was taken as 1,000 colonoscopies per year may not represent the true incidence of IBD in the general population. Conducting a population-based study would significantly enhance our understanding of various aspects of inflammatory bowel disease. CD involving the bowel proximal to the terminal ileum might have been missed during colonoscopy, leading to an underestimation of the true incidence of CD.

## Conclusions

The incidence of IBD is quite high as shown by this study. Increasing awareness of the disease, improved diagnostic modalities, urbanization, and lifestyle change may be responsible for its incidence and detection. UC was predominantly diagnosed with CD. Females were more commonly affected than males. Most of the patients belonged to the young and middle-aged population. The majority of patients with UC had extensive colitis. Similarly, most patients with CD were ileo-colonic in location with non-stricturing and non-penetrating phenotypes. Arthralgia and arthritis were the commonest extra-intestinal manifestations. Patients were mostly treated with conventional therapy including mesalamine, prednisolone, and azathioprine. The use of biologics was fairly low. The cost and availability of biologics/biosimilars are of utmost concern. There is a need to establish an IBD registry system in the country.
